# Hypomagnesemia in short bowel syndrome patients

**DOI:** 10.1590/S1516-31802000000600004

**Published:** 2000-11-01

**Authors:** Simone Chaves Miranda, Michelle Lizzy Bandeira Ribeiro, Eduardo Ferriolli, Júlio Sérgio Marchini

**Keywords:** Hypomagnesemia, Small gut syndrome, Intestine. Nutrition support, Hipomagnesemia, Síndrome do Intestino Curto, Intestino, Suporte Nutricional

## Abstract

**CONTEXT:**

Magnesium support to small bowel resection patients.

**OBJECTIVE:**

Incidence and treatment of hypomagnesemia in patients with extensive small bowel resection.

**DESIGN:**

Retrospective study.

**SETTING:**

Metabolic Unit of the University Hospital Medical School of Ribeirão Preto, University of São Paulo, Brazil.

**PATIENTS:**

Fifteen patients with extensive small bowel resection who developed short bowel syndrome.

**MAIN MEASUREMENTS:**

Serum magnesium control of patients with bowel resection. Replacement of magnesium when low values were found.

**RESULTS:**

Initial serum magnesium values were obtained 21 to 180 days after surgery. Hypomagnesemia [serum magnesium below 1.5 mEq/l (SD 0.43)] was detected in 40% of the patients [1,19 mEq/l (SD 0.22)]. During the follow-up period, 66% of the patients presented at least two values below reference (1.50 mEq/l). 40% increased their serum values after magnesium therapy.

**CONCLUSION:**

Metabolic control of serum magnesium should be followed up after extensive small bowel resection. Hypomagnesemia may be found and should be controlled.

## RESUMO

**CONTEXTO:**

Suporte nutricional de magnésio em pacientes com ressecção de intestino curto.

**OBJETIVO:**

Analisar a incidência e tratamento de hipomagnesemia em pacientes com pequena ressecção extensiva do intestino.

**TIPO DE ESTUDO:**

Estudo retrospectivo.

**LOCAL:**

Unidade Metabólica Hospital das Clínicas da Faculdade de Medicina de Ribeirão Preto - Universidade de São Paulo.

**PACIENTES:**

15 pacientes pós grande enterectomia evoluindo com Síndrome do Intestino Curto.

**VARIÁVEIS ESTUDADAS:**

Controle sérico de magnésio de pacientes com ressecção intestinal. Reposição de magnésio quando valores baixos foram encontrados.

**RESULTADOS:**

Valores séricos de magnésio iniciais [1,59 mEq/l (SD 0,43)] foram obtidos 21 a 180 dias após a cirurgia. Hipomagnesemia (magnésio sérico menor do que 1,5 mEq/l) foi detectada em 40% dos pacientes [1,19 mEq/l (SD 0,22)]. Durante o período de seguimento, 66% dos pacientes apresentaram pelo menos dois valores abaixo dos limites de referência. 40% aumentaram seus valores após terapia com magnésio.

**CONCLUSÃO:**

O controle metabólico do magnésio sérico deve ser feito após enterectomia extensa. Hipomagnesemia pode ser encontrada e deve ser controlada.

## INTRODUCTION

Magnesium is the fourth most abundant cation in the blood. Its existence in animals was detected around 1859. Magnesium deficiency was described in the early 1930s. Magnesium depletion has been found in alcoholics and other patients after long hospitalization.^1,2,3^ The human body has 25 to 30 grams of magnesium, but values of up to 40 g have been described. It is concentrated in bones, which contain about 24 g (50 to 60% intracellular); 1% of the total body magnesium pool is found in the blood plasma (50% free and 1/3 bound to albumin).^1^

The Recommended Dietary Allowance (RDA) for magnesium ranges from 280 to 350 mg or 23 to 30 mEq/day.^4^ Magnesium absorption occurs all over the entire gastrointestinal tract, but mainly in the ileum and colon. There is evidence to support the secretion of this ion by the duodenum. Cellular magnesium transport occurs through active and passive mechanisms and is inhibited by calcium, alcohol, phosphate, phytates and fat, and stimulated by vitamin D. Renal excretion is inversely proportional to extracellular concentration.^2,5^ The important role of magnesium in human metabolism can be recognized through its functions as a stabilizer of ATP-depen- dent enzymatic reactions, as a cofactor of *circa* 300 enzymes, as a modulator in neuromuscular transmission and as an essential ion in cardiac physiology.^2,6^ Its action on the myocardium during ischemic episodes and reperfusion injury after acute myocardium infarction have been shown in studies such as the Second Leicester Intravenous Magnesium Intervention Trial (LIMIT-2).^7^ It has also been shown to have beneficial effects in the treatment of cardiac arrythmia.^8,9^ A link between hypomagnesemia and sudden death has been strongly suggested and also its correlation with higher numbers of ischemic episodes or higher intake of nitrates in ischemic patients.^8,9^ There is an inverse relationship between magnesemia, deaths and hospital care following vascular or coronary disease^10,11,12^. Magnesium deficiency can also lead to premenstrual tension syndrome and depression as a result of diminished dopamine synthesis. It is one of the micronutrients related to fetal malformation in laboratory animals, either as an excess or deficiency.^2,6^ Hypomagnesemia is common among patients who need intensive care and in the general population.^3,6^

Magnesium administration must be done carefully for patients with severe atrioventricular block and renal insufficiency, as high levels of this ion may decrease the cardiac rate.^7^ Food sources of magnesium are vegetables, roots, seafood, nuts, cereals and milk products. Drinking water offers 1 - 16 ppm. This cation is part of chlorophyll and so is present in all green plants. Magnesium may be lost during cooking. To retain this micronutrient one should cook food in small amounts of water and for the shortest time possible.^1,5^

This study had the aim of examining hypomagnesemia incidence among patients with extensive small bowel resection, and following up these patients during their hospitalization and treatment of their deficiency.

## METHODS

Fifteen patients from the Metabolic Unit of the University Hospital of the Medical School of Ribeirão Preto - Universidade de São Paulo, Brazil, were retrospectively reviewed concerning their magnesium status. All were submitted to extensive small bowel resection (> 2 meters) and their small bowel transit time was less than ten minutes. None of the studied patients had any documented intestinal disease before surgery. The cause of enterectomy in all patients was intestinal ischemia, either thrombosis or emboli. Among these 15 patients, 7 also had colectomy, at least of the ascending colon. The most extensive resection was of the ascending colon and transverse colon. Values of their first magnesium serum levels after surgery, the follow-up of serum data during hospitalization and treatment of the deficiency were analyzed. To treat the deficiency, at least twice the RDA values (23 to 30 mEq) were given by intravenous infusion, i.e. 10 ml of magnesium sulfate 20%. The serum level of magnesium for eutrophic subjects is 1.5 to 2.5 mEq/l.^2^

## RESULTS

**Figure 1:**
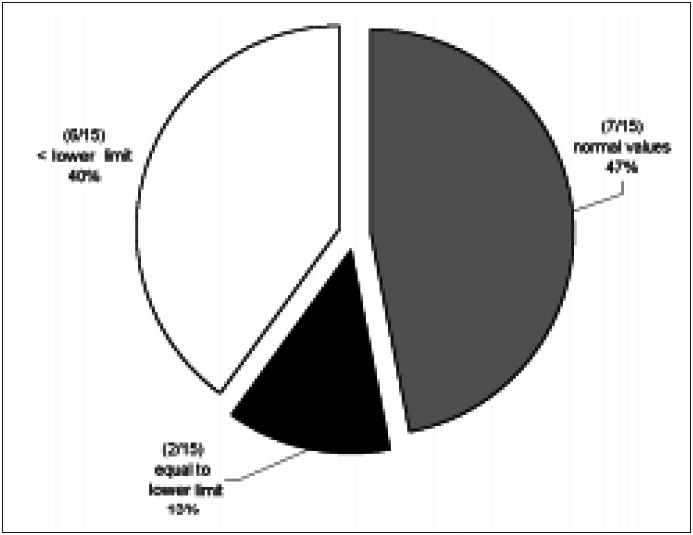
Percentage incidence of hypomagnesemia in the first magnesium determination after surgery.

**Figure 2:**
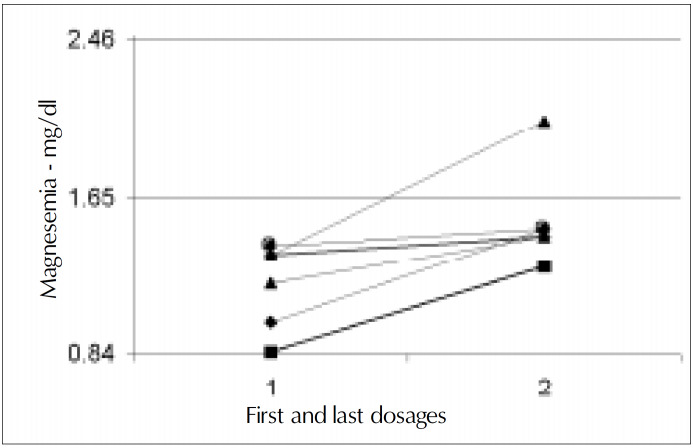
Evolution of serum magnesium value in patients with hypomagnesemia. Initial value = 1.19 mEq/l (SD 0.22). End value = 1.52 mEq/l (SD 0.26).

**Figure 3:**
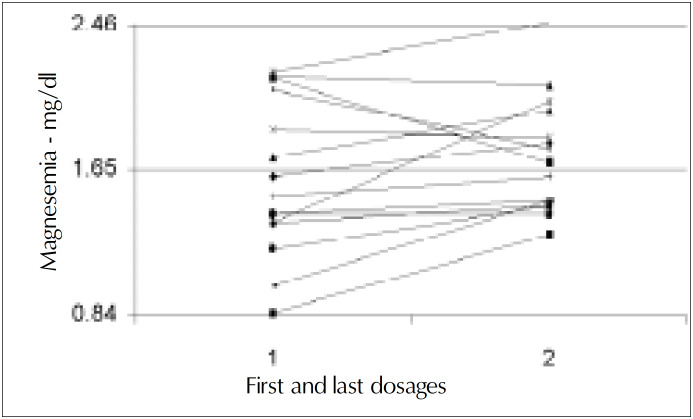
Evolution of magnesemia in all patients. Initial value = 1.59 mEq/l (SD 0.43). End value = 1.72 mEq/l (SD 0.33).

Initial plasma magnesium values [mean 1.59 mEq/l (SD 0.43)] were obtained from 21 to 180 days after surgery, 45 days on average. Magnesemia in the first collection varied from 0.85 to 2.17 mEq/l and was low in 6 (40%, 1.19 mEq/l SD 0.22) patients out of 15. Two patients (13.3%) had magnesium plasma levels at the lower limit *(Figures 1, 2 and 3)*. There was no relationship between resection of the colon and hypomagnesemia. Patients with normal magnesemia were on average monitored on the twentieth postoperative day, i.e. earlier than the others. Of those eight patients who had magnesemia equal to or less than the lower dosage limit, only two received supplements when diagnosis was known. During hospitalization with nutritional support therapy, 10 out of the 15 patients followed showed serum magnesium below the lower limit, on at least two occasions. From these, only four received specific treatment for hypomagnesemia. No patient presented hypermagnesemia.

## DISCUSSION

The status of serum magnesium after large bowel resection surgery may affect patients’ treatment and follow-up. Hypomagnesemia can be found just after surgery or during follow-up of such patients. A sequence of low values of serum magnesium was found in 10 out of our 15 patients, under our continuous long term study program of these patients. It is important to recognize that prolonged parenteral nutrition alone would not explain the fall of magnesium levels, as the patient received the recommended daily allowances.^4^ Magnesium nutrition control has been shown to be inadequate, causing patients to face micronutrient imbalance and suffer its consequences. In spite of problems in correlating blood magnesium levels to their intracellular amounts, plasma values could help to detect or at least suggest body magnesium deficiency and the need for treatment.

Our present and suggested magnesium treatment for these patients is: a) when serum levels range from 1.2 to 1.5 mEq/l they should receive a total infusion of 66 mEq/day, 20 milliliters of magnesium sulfate 20% plus daily allowances, diluted in 100 or 200 ml saline or glucose solution, depending on the patient's liquid restrictions; b) patients with lower magnesium levels (< 1.2 mEq/l), should receive an infusion of 99 mEq per day plus allowances, keeping the dilution as described above.

Infusion should be at a low rate like 5 mEq per hour (side effects have been reported when magnesium sulfate is infused rapidly i.e. paresthesia, nausea, vomiting, malaise and hypotension). In our unit, we check magnesium blood levels 24 hours after reposition, and a week later if the procedure was efficient. After the patient has been stabilized, we continue checking it every 15 days during hospitalization and monthly, when the patient is at home.

In conclusion, it can be said that low values of magnesium serum were found in patients undergoing resection of the small intestine. Levels of blood magnesium must be controlled when patients are kept on parenteral nutrition for long periods, even when receiving the recommended daily allowances of the ion, to have better control of their desirable or adequate nutritional status concerning this micronutrient.

## References

[1] Dutra-de-Oliveira JE, Marchini JS (1998). Ciências Nutricionais.

[2] Shils ME, Olson JA, Shike M (1994). Modern Nutrition in: Health and Disease.

[3] Britton J, Pavord I, Richards K (1994). Dietary magnesium, lung function, wheezing, and hyper-reactivity in a random adult population sample. Lancet.

[4] National Research Council (1989). Food and Nutrition Board. Recommended Dietary Allowances: 10^th^ revised Edition.

[5] Mahan LK, Arlin MT (1995). Krause Alimentos, Nutrição e Dietoterapia.

[6] McLean RM (1994). Magnesium and its therapeutic uses: a review. Am J Med.

[7] Woods KL, Fletcher S (1994). Long-term outcome after intravenous magnesium sulfate in suspected acute myocardial-infarction - The Second Leicester Intravenous Magnesium Intervention Trial (LIMIT-2). Lancet.

[8] Eisenberg MJ (1992). Magnesium deficiency and sudden death (editorial). Am Heart J.

[9] Lasserre B, Spoerri M, Moullet V, Theubet MP (1994). Should magnesium therapy be considered for the treatment of coronary heart disease? II. Epidemiological evidence in outpatients with and without coronary heart disease. Magnes Res.

[10] Bernardi D, Dini FL, Azzarelli A, Giaconi A, Volterrani C, Lunardi M (1995). Sudden cardiac death rate in an area characterized by high incidence of coronary artery disease and low hardness of drinking water. Angiology.

[11] Gartside PS, Glueck CJ (1995). The important role of modifiable dietary and behavioral characteristics in the causation and prevention of coronary heart disease hospitalization and mortality: the prospective NHANES I follow-up study. J Am Coll Nutr.

[12] Altura BM, Zhang A, Altura BT (1993). Magnesium, hypertensive vascular diseases, atherogenesis, subcellular compartmentation of Ca^2+^ and Mg^2+^ and vascular contractility. Miner Electrolyte Metab.

